# A huge benign fibrous histiocytoma arising from the renal capsule: report of a case

**DOI:** 10.1186/s12882-019-1256-7

**Published:** 2019-02-21

**Authors:** Chao Sun, Shengli Wang, Bo Li, Xueting Sun

**Affiliations:** 1 Department of Urology, The third the People’s Hospital of Bengbu, 38# Shengli Road, Bengbu, Anhui Province China; 2 Department of Nursing, The third the People’s Hospital of Bengbu, 38# Shengli Road, Bengbu, Anhui Province China

**Keywords:** Benign fibrous histiocytoma, Renal capsule, Renal neoplasms

## Abstract

**Background:**

Neoplasms originating in the renal capsule are very rare. Benign fibrous histiocytoma(BFH) most commonly occurs in the dermis and subcutis, few cases of this tumor appear in the renal capsule. In particular, BFH larger than 20 cm are scarce. Here we report a rare huge one measuring 23 × 13 × 7 cm.

**Case presentation:**

We report a 64-year-old man who presented with a few-months history of dull pain in the right groin. The tumor had its point of origin in the renal capsule which is a rare condition. Histologically, the tumor was composed of intersecting fascicles of fibroblastic cells forming a “storiform” pattern. Immunohistochemical studies were also performed, ultimately leading to the diagnosis of BFH. The patient was treated with radical nephrectomy. No recurrence was detected 4 months after surgery.

**Conclusions:**

BFH arising from the renal capsule was very rare. In particular, the case of more than twenty centimeters is extremely rare. The clinical presentation of renal BFH might be only a mass. However, differential diagnosis from renal cell carcinoma proved to be impossible before surgical intervention. It is difficult to diagnose only by means of histopathology, but the immunohistochemical method can provide a clear and definite diagnosis.

## Background

Benign fibrous histiocytoma(BFH) most commonly occurs in the dermis and subcutis, deeply-situated counterparts originating in bone [1] and deeper soft tissue have been described but less common than cutaneous ones [2]. This tumor rarely occurs in the parenchyma organs [3, 4], and Shoji K. et al. first reported a case of BFH originating in the renal capsule in 1992 [5], measuring 6 × 4.5 × 6 cm. In this paper we report a huge BFH measuring 23 × 13 × 7 cm, and the clinical picture and pathological findings are described.

## Case presentation

**Fig. 1:**
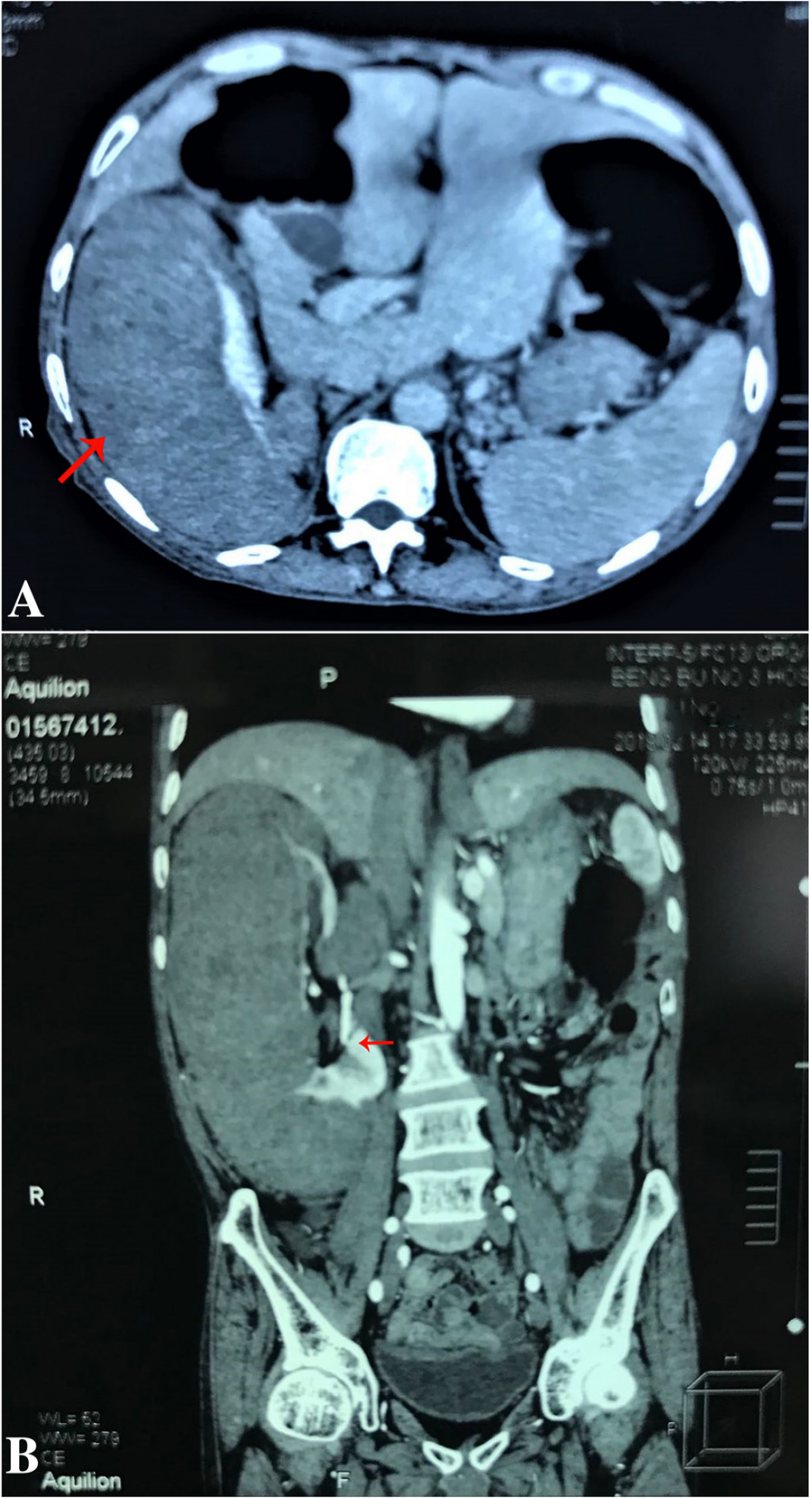
**a** CT scan demonstrates a large solid mass of the right kidney about 20 cm in diameter (arrow); **b** Renal arteriography demonstrated a hypovascular tumor and compressed deformity of pelvis of the right kidney (arrow)

A 64-year-old man was hospitalized for a few-months history of dull pain in the right groin. Physical examination revealed a palpable mass in the right flank with a mild right flank tenderness. His previous history was uneventful. The results of laboratory examination were unremarkable. Screening ultrasound examination revealed a hypoechoic tumor with inhomogeneous interior echoes, 23 × 13 × 7 cm in size. Computerized tomography (CT) confirmed the presence of a solid tumor of the right kidney about 20 cm in diameter (Fig. 1a, arrow). Renal arteriography demonstrated a hypovascular tumor and compressed deformity of pelvis of the right kidney (Fig. 1b, arrow). The left kidney was normal. Suspecting a renal carcinoma the patient underwent a right radical nephrectomy in February 2018. He had an uneventful post-operative recovery, and is currently well without any sign of recurrence.

**Fig. 2:**
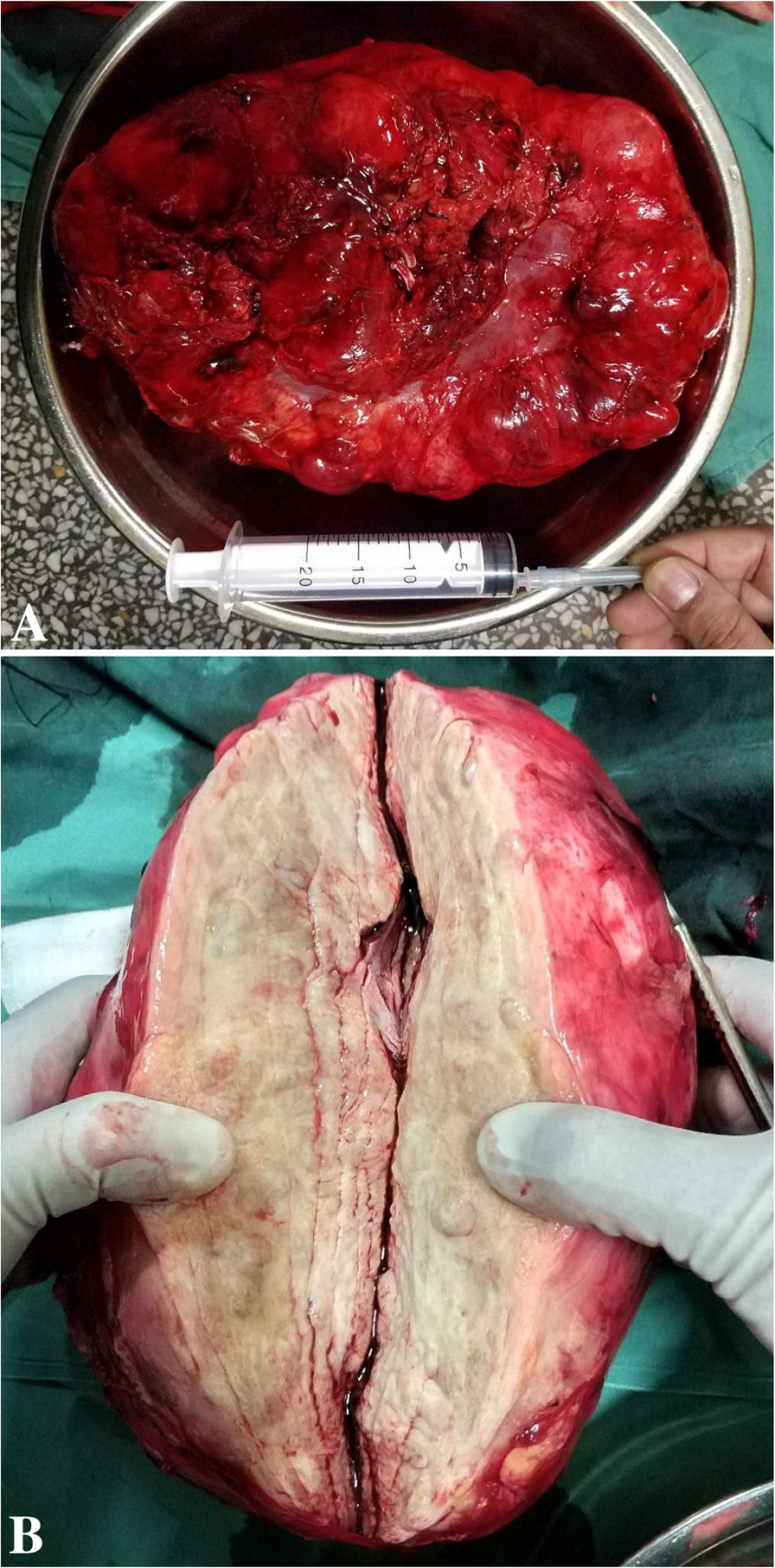
**a** The tumor was grossly solitary and sharply defined, measuring 23 × 13 × 7 cm; **b** The cut surface was solid, elastic hard, and white yellowish without hemorrhage or necrosis

**Fig. 3:**
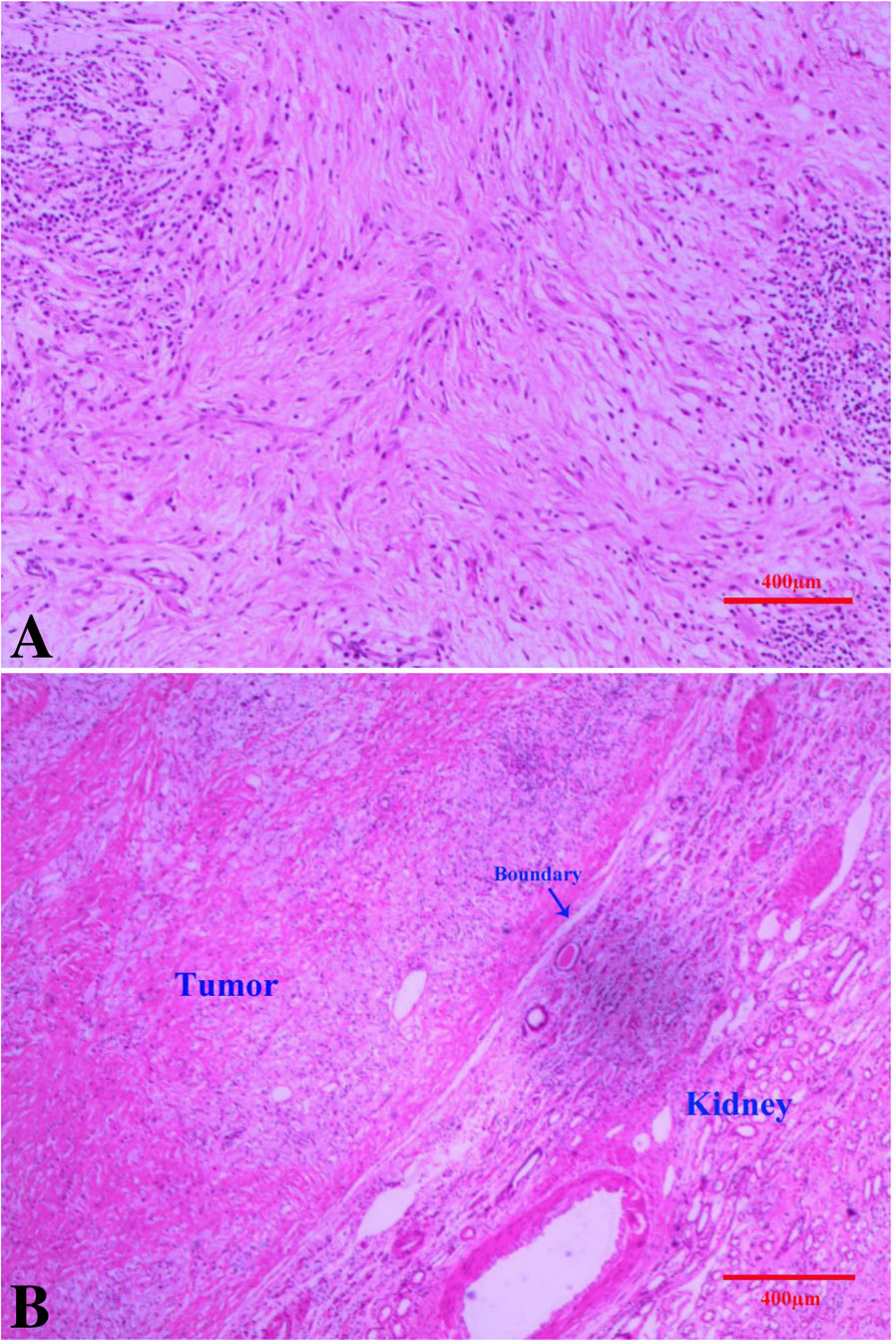
**a**: The tumor disclosed intersecting fascicles of fibroblastic cells forming a “storiform” pattern; **b**: There was a clear boundary between the tumor and the kidney tissue under the microscope. HE. × 100

Grossly, the tumor was solitary and sharply defined, measuring 23 × 13 × 7 cm (Fig. 2a). The cut surface was solid, elastic hard, and white yellowish without hemorrhage or necrosis (Fig. 2b). The pyelocaliceal system and the renal vessels were free of tumor involvement. Microscopic examination of the tumor disclosed intersecting fascicles of fibroblastic cells forming a loose crisscross or “storiform” pattern (Fig. 3a). And we observed that there was a clear boundary between the tumor and the kidney tissue under the microscope, which kept in line with the CT representation (Fig. 3b). The tumor was basically histiocytic, presenting a great deal of collagenic fibers, upon the presence of foam cells. The multinucleated giant cells and undifferentiated mesenchymal cells were very low. In addition, the cells were well differentiated, and the nuclei were not deep stained with very low heteromorphism.

Immunohistochemical studies showed that the tumor cells were strongly and positive for CD34, vimentin and CD99, the proliferation index of Ki-67 was 1%, but were negative for the smooth muscle actin (SMA), the desmin, SOX10, Bcl-2, CK7, the melanogenesis marker (HMB45), MelanA and the S-100 protein.

## Discussion and conclusions

Neoplasms originating in the renal capsule are very rare. Among fibrohistiocytic tumors, BFH [5] and malignant fibrous histiocytoma(MFH) [6, 7] have been described in the literature. Shoji K. et al. first reported a case of BFH originating in the renal capsule in 1992 [5], measuring 6 × 4.5 × 6 cm. Here we report a huge one measuring 23 × 13 × 7 cm.

BFH most common arise from the dermis. There have been a few cases reported that this tumor appeared in parenchymal organs, such as the kidney [4] lung [3], and orbit [8, 9]. The BFH we describe had its point of origin in the renal capsule outside the kidney. The tumor was well circumscribed by thick connective tissue and had no infiltrating features. Expanding from this place, the tumor displaced the kidney without involving the renal parenchyma. The results we reported were consistent with Shoji K. et al. reported [5]. Otherwise, in the first reported case of BFH in the kidney which was not from the renal capsule [8], the tumor occupied the middle pole of the kidney and compressed the lower and middle portion of the renal pelvis.

BFH included fibroxanthoma, atypical fibroxanthoma, dermatofibroma and sclerosing hemangioma. All of these tumors showed mainly histiocytic properties, but they vary depending on the amount of collagen fibers, or on the existence of foam cells, capillaries, multinucleated giant cells and hemosiderin [10]. In our case tumor cells showed mainly fibroblastic properties consisting of a storiform arrangement. Furthermore, the tumor presented a great deal of collagenic fibers, upon the presence of foam cells. The multinucleated giant cells and undifferentiated mesenchymal cells were very low. The cells were well differentiated and the nuclei were not deep stained with very low heteromorphism.

MFH is the most frequently subtype of soft-tissue sarcoma in late adulthood in both sexes and left renal predilection [11, 12]. Approximately one third of MFH occurs in the extremities [13]. Retroperitoneum or kidney origin accounts for 12–14% of all cases [14]. There are few reported cases of MFH definitely arising from the renal capsule [6, 7, 15, 16]. The histology characters of MFH were groups of histiocytes and pleomorphic giant cells with large nuclei and moderate mitotic activity. And the prognosis of MFH is different from BFH. Despite extensive surgical excision, MFH shows a very poor prognosis for local recurrence or distant metastasis. Sixty percent of patients died of the disease within 1 year of surgery [12, 17].

The majority diagnostic imaging of BFH and MFH included ultrasound and CT. Ultrasound in most of the cases showed a well-defined mass with a rather complex internal pattern. In our case this method showed a spherical well defined echogenic mass. CT showed a solid mass with or without necrotic areas. However, it cannot be differentiated radiologically from renal cell carcinoma. Thus, treatment selection was no more than a radical nephrectomy because of suspicion of renal cell carcinoma. We performed a transperitoneal nephrectomy, and the patient is currently well without any sign of recurrence.

It is difficult to diagnose by histopathological methods, whereas immunohistochemistry can provide a relatively correct diagnosis [18]. In this case, immunohistochemical staining of various elements was of assistance. Some researchers recommend the use of electron microscopy [19], but other researchers believe that the effect of electron microscopy is limited because it does not show specific features of epithelial and histiocytic features [20].

BFH arising from the renal capsule was very rare. In particular, the case of more than twenty centimeters is extremely rare. The clinical presentation of renal BFH might be only a mass. However, differential diagnosis from renal cell carcinoma proved to be impossible before surgical intervention. It is difficult to diagnose only by means of histopathology, but the immunohistochemical method can provide a clear and definite diagnosis.

## Abbreviations

BFH: Benign fibrous histiocytoma
CT: Computerized tomography
MFH: Malignant fibrous histiocytoma
SMA: Smooth muscle actin
